# Multifactorial Causes of Chronic Mortality in Juvenile Sturgeon (*Huso huso*)

**DOI:** 10.3390/ani10101866

**Published:** 2020-10-13

**Authors:** Sara Ciulli, Enrico Volpe, Rubina Sirri, Giorgia Tura, Francesca Errani, Gianpiero Zamperin, Anna Toffan, Marina Silvi, Andrea Renzi, Miriam Abbadi, Lorena Biasini, Tobia Pretto, Pietro Emmanuele, Antonio Casalini, Giuseppe Sarli, Patrizia Serratore, Oliviero Mordenti, Luciana Mandrioli

**Affiliations:** 1 Department of Veterinary Medical Sciences, University of Bologna, 40064 Bologna, Italy; sara.ciulli@unibo.it (S.C.); enrico.volpe2@unibo.it (E.V.); rubina.sirri2@unibo.it (R.S.); giorgia.tura3@unibo.it (G.T.); francesca.errani2@unibo.it (F.E.); marina.silvi@unibo.it (M.S.); andrea.renzi6@unibo.it (A.R.); pietro.emmanuele@unibo.it (P.E.); antonio.casalini6@unibo.it (A.C.); giuseppe.sarli@unibo.it (G.S.); patrizia.serratore@unibo.it (P.S.); oliviero.mordenti@unibo.it (O.M.); 2 Istituto Zooprofilattico Sperimentale delle Venezie, Legnaro, 35020 Padua, Italy; gzamperin@izsvenezie.it (G.Z.); atoffan@izsvenezie.it (A.T.); mabbadi@izsvenezie.it (M.A.); lbiasini@izsvenezie.it (L.B.); tpretto@izsvenezie.it (T.P.)

**Keywords:** *Huso huso*, sturgeon, chronic mortality, environmental stressors, nutritional imbalance, bacterial septicemia, metagenomic analysis, bioinformatics, de novo assembly, *Herpesvirales*, lymphohematopoietic pathology

## Abstract

**Simple Summary:**

Sturgeons are species of biological and economic importance. Among them, the beluga sturgeon (*Huso huso*, Linnaeus, 1758) is the largest freshwater fish and can reach a maximum weight of 1000 kg. Over 80% of existing sturgeon species are endangered, vulnerable, or on the brink of extinction because of their late sexual maturity and long periods between spawning in the wild. Many species of sturgeon are being reared to increase natural populations or produced for consumption as human food. Despite considerable global interest in sturgeon aquaculture, there is still a paucity of information on nutrient requirements and utilization and even their general nutrition. The development of sturgeon aquaculture has been accompanied by an increase in disease outbreaks, representing a serious problem. This paper presents an investigation of chronic mortality outbreak. The clinical presentation of diseased sturgeons, which is suggestive of a neurologic condition, was further investigated using various laboratory analyses that excluded the presence of the main viral agents of sturgeons while confirming the presence of both bacteria and mycetes. The pathological findings point out the severe myopathy, depletion of lymphohematopoietic tissue, and signs of degeneration consistent with a suboptimal environmental state. Results from chemical analyses also suggest nutritional imbalance.

**Abstract:**

This investigation focused on an episode of chronic mortality observed in juvenile *Huso huso* sturgeons. The examined subjects underwent pathological, microbiological, molecular, and chemical investigations. Grossly severe body shape deformities, epaxial muscle softening, and multifocal ulcerative dermatitis were the main observed findings. The more constant histopathologic findings were moderate to severe rarefaction and disorganization of the lymphohematopoietic lymphoid tissues, myofiber degeneration, atrophy and interstitial edema of skeletal epaxial muscles, and degeneration and atrophy of the gangliar neurons close to the myofibers. Chemical investigations showed a lower selenium concentration in affected animals, suggesting nutritional myopathy. Other manifestations were nephrocalcinosis and splenic vessel wall hyalinosis. Septicemia due to bacteria such as *Aeromonas veronii*, *Shewanella putrefaciens*, *Citrobacter freundii, Chryseobacterium* sp., and pigmented hyphae were found. No major sturgeon viral pathogens were detected by classical methods. Next-generation sequencing (NGS) analysis confirmed the absence of viral pathogens, with the exception of herpesvirus, at the order level; also, the presence of *Aeromonas veronii* and *Shewanella putrefaciens* was confirmed at the family level by the metagenomic classification of NGS data. In the absence of a primary yet undetected biological cause, it is supposed that environmental stressors, including nutritional imbalances, may have led to immune system impairment, facilitating the entry of opportunistic bacteria and mycotic hyphae.

## 1. Introduction

Wild sturgeon populations have undergone a dramatic decline due to legal and illegal overfishing, habitat deterioration, river fragmentation including damming, and pollution. Since the end of the 20th century, sturgeon farming has partially replaced meat and caviar production from fisheries. Now, *Acipenseridae*, including the species *Huso huso*, are reared for different purposes, depending on the commercial supply, for meat and caviar production [1].

The development of sturgeon aquaculture activities has been accompanied by the occurrence of disease outbreaks, and the possibility of the emergence and rapid dissemination of several infectious disease agents represents a serious problem in sturgeon aquaculture [2]. Several viral, bacterial, fungal, and parasitic diseases have been reported worldwide. Due to the limited knowledge about epizootiology and disease control methods, infectious diseases may represent a major challenge in sturgeon aquaculture. Moreover, none of the diseases reported in sturgeon are regulated by the World Organization for Animal Health (OIE) or European Union (EU) legislation [2]. It is believed that sturgeons are comparatively more resistant to disease than other fish species; nevertheless, many studies have shown that their diseases involve different pathogens. Intensive rearing exposes the fish to several sources of stress, such as high stock density and manipulation that predispose them to a number of infectious diseases associated with viral or bacterial pathogens [3]. As in the farming of other fish species, diseases are a principal limiting factor in sturgeon farming. Among them, viral diseases often cause major damage to the industry. Disease control in sturgeon farming is difficult due to a lack of knowledge about disease epidemiology and diagnostic methods [4]. As a result of more intensive sturgeon aquaculture, infectious diseases that affect sturgeon species have emerged [2].

Recent mortality outbreaks in sturgeons were described in association with viruses such as nucleocytoplasmic large DNA viruses (NCLDVs) [4,5,6] and herpesviruses [7,8], *Veronaea botryosa* phaehyphomycoses [9], and bacteria. Particularly, in *H. huso* and hybrids, bacterial infections caused by *Vibrio vulnificus*, *Lactococcus lactis* subsp. *lactis*, *Aeromonas hydrophila*, and *Yersinia ruckeri* are reported [10,11]. The aim of this study was to describe an episode of chronic mortality in juvenile *Huso huso* in order to expand upon and add more detail to what has been preliminarily reported by Ciulli et al. [12] using a multidisciplinary approach that examines each aspect through numerous technical investigations.

## 2. Materials and Methods

### 2.1. Animals

**Figure 1:**
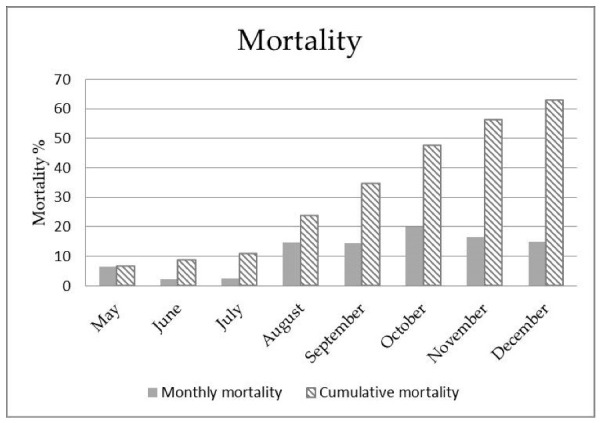
Monthly and cumulative mortality rates observed in *Huso huso* outbreak.

Thirteen *Huso huso* juveniles consisting of 11 diseased and 2 aged-matched animals were investigated in this study. The 11 diseased juveniles belonged to a group of 46 fingerlings kept in a recirculating aquaculture system with a water temperature of 19 °C. Since spring 2018, some animals started to show abnormal swimming behavior. The main clinical manifestations were neurologic in nature, and they included circular swimming, sudden movements, and hyperactivity to stimuli alternating with prolonged resting on the bottom or lying on the side (Video S1). Chronic “dripping” mortality lasted several months until December 2018 (Figure 1).

### 2.2. Pathological Sampling

Celomatic effusions were sampled for cytology. Direct and sediment smears after centrifugation were prepared and May–Grünwald–Giemsa stained. To detect the presence of microorganisms, some slides were also stained with periodic acid–Schiff (PAS) stain, which reacts with the cell walls.

Main organs (multiple transverse sections of the body, liver, intestine, spleen, heart, head, and gills) were sampled for routine histology. The sections of the body containing the vertebral segments and tissues with cartilage and bone were placed in mild decalcifier solution (Osteodec, Bio-optica, Italy) for a few hours. From the paraffin blocks, 3 µm thick sections were cut and hematoxylin and eosin (H&E) stained. Gram, Giemsa, Ziehl–Neelsen, and PAS stain were also used to highlight intralesional etiological agents.

### 2.3. Virological Investigation

Brain, kidney, spleen, gill, and skin samples were taken for virological investigation. RNA was extracted from about 25 mg of brain tissue using a NucleoSpin RNA kit (Macherey-Nagel, Duren, Germany) according to the manufacturer’s instructions. The RNA was used soon after extraction or stored at −80 °C until use. The extracts were subjected to reverse transcriptase polymerase chain reaction (RT-PCR) analysis to detect the presence of betanodaviruses using previously described protocols [13,14].

DNA was extracted from about 20 mg tissue samples composed of viral target organs (pools of internal organs and gills for adenoviruses or herpesviruses, and pools of skin and gills for nucleocytoplasmic large DNA viruses) using the PureLink Genomic DNA kit (Invitrogen, Carlsbad, CA, USA) following the manufacturer’s instructions. The DNA was used soon after extraction or stored at −20 °C until use. The extracted DNA was subjected to selected PCR analysis with Taq polymerase (Invitrogen, Carlsbad, CA, USA) in order to detect adenoviruses, herpesviruses, and nucleocytoplasmic large DNA viruses (NCLDVs) that have previously been reported in sturgeons following previously described protocols [4,5,15,16,17].

Viral cultivation was performed on pooled samples of spleen, kidney, heart, and gills. Samples were ground with sterile sand quartz in sterile cooled mortars, diluted 1:10 in L-15 supplemented with 10% fetal bovine serum, 1% antibiotic/antimycotic solution containing 10,000 IU/mL penicillin G, 10 mg/mL streptomycin sulfate, 25 g/mL amphotericin B, and 0.4% 50 mg/mL kanamycin solution, and centrifuged at 4000× *g* at 4 °C for 15 min. Supernatants were collected and kept at 4 °C overnight to give the antibiotics time to inhibit bacterial growth. Thereafter, supernatants were inoculated onto 24 h old white sturgeon skin cell line (WSSK) and incubated at 20 °C. Cultivation was done for 7 days, followed by 2 blind passages.

### 2.4. Bacteriological Examination

Kidney, spleen, brain, and skin lesions, if present, were sampled by loop and immediately streaked onto tryptone soy agar (TSA; Oxoid, Basingstoke, UK). Plates were incubated at 20 °C for 3 days. The material from a few randomly chosen dominant colonies was inoculated onto TSA and incubated at 20 °C for 24 h to obtain pure cultures. Colonies were Gram-stained for cell morphology determination. Then, molecular identification through 16S rDNA amplification and sequencing was conducted. Briefly, DNA was extracted from pure colonies through the boiling method, and the amplification of 16S rDNA was performed with primers P0F and P6R using 100 µL of each lysed cell suspension according to a previously described procedure [18]. When *Aeromonas* sp. was suspected, species identification was conducted by *gyr*B gene amplification according to a previously described protocol [19] and sequencing. Polymerase chain reaction products were purified and sequenced for bacterial identification. Sequences were obtained through the Bio-Fab Sequencing Service (Rome, Italy) and then analyzed using the online Basic Local Alignment Search Tool (BLAST) web interface provided by the National Center for Biotechnology Information (NCBI) to confirm bacterial identity [20].

### 2.5. Chemical Investigations

Selenium concentration (expressed in mg/kg) was analyzed in the frozen hearts of 2 diseased and 2 age-matched animals. Sample mineralization was performed by adding 6 mL of nitric acid (67% purity; Ultrapure Merck, Darmstadt, Germany) through an Ethos One Milestone microwave (Milestone S.r.l., Sorisole, Italia) following the 3052 US EPA [21] method. Then, digested samples were diluted to 15 mL with 18 mΩ Milli-Q ultrapure water. The detection of selenium was obtained by means of an Optima 2100 inductively coupled plasma atomic emission spectrometer (ICP–OES; PerkinElmer, Waltham, MA, USA) coupled with a CETAC U5000 ultrasonic nebulizer (Thermo Fisher Scientific, Waltham, MA, USA) in axial view configuration following the 6010c US EPA [22] method. The quality of data was verified using the DORM-4 dogfish muscle certified reference material for trace metals (National Research Council of Canada, Ottawa, ON, CA). Selenium concentration values were compared through *t*-test analysis (Prism version 6.0 software, GraphPad Software, San Diego, CA, USA).

### 2.6. Next-Generation Sequencing (NGS): DNA Extraction, Library Preparation, and Sequencing

Total DNA was purified from the diseased brain tissue of a single *H. huso* using the QIAamp DNA Mini Kit^®^ (Qiagen) according to the manufacturer’s instructions. DNA concentration was measured using a Qubit^®^ dsDNA HS Assay Kit with the Qubit™ 4 Fluorometer (Thermo Fisher Scientific) and stored at –20 °C. Purified DNA was used to prepare the sequencing library employing a KAPA HyperPlus Kit (Roche); Agencourt AMPure XP (Beckman Coulter) was used for fragment selection. Fragment quality and size were checked with the Agilent High-Sensitivity DNA kit on Agilent 2100 Bioanalyzer System (Agilent Technologies) and normalized by adjusting molar concentration. The library was sequenced in paired-end mode with NextSeq^®^ 500/550 Mid Output Kit v2.5 (300 cycles) using the NextSeq™ 550 Sequencing System (Illumina).

### 2.7. NGS: Bioinformatics Analysis

Illumina reads quality was assessed using FastQC v0.11.2 [23]. Raw data were filtered by removing (a) reads with more than 10% of undetermined (N) bases, (b) reads with more than 100 bases with a Q score below 7, and (c) duplicated paired-end reads. Remaining reads were clipped from Illumina adaptor TruSeq with Scythe v0.991 (https://github.com/vsbuffalo/scythe) and from the PCR primer with Trimmomatic v0.32 [24] before being trimmed with Sickle v1.33 [25]. Reads shorter than 50 bases or unpaired after previous filters were discarded.

Taxonomic assignment of high-quality reads was carried out using BLAST 2.7.1+ [26] alignment against the integrated NT database (version 12 February 2018) and diamond 0.9.17 [27] alignment against the integrated NR database (version 12, February 2018). Alignment hits with e-values greater than 1 × 10^−3^ were filtered out. The taxonomic level of each read was determined using the lowest common ancestor (LCA) algorithm implemented in MEGAN UE v6.10.8 [28]. Reads taxonomically classified as belonging to *Herpesvirales* order were selected and de novo assembled using IDBA-UD v1.1.1 [29] with the multi-kmer approach using a minimum value of 24, a maximum value of 124, and an inner increment of 5. Resulting contigs were manually classified using the NCBI BLAST online suite [20] at both the nucleotide and amino acid level with the blastn and blastx algorithm, respectively.

### 2.8. PCR Confirmation of NGS Data

In order to confirm the data obtained by next-generation sequencing (NGS) and further investigate the presence of a putative herpesvirus sequence in diseased and aged-matched subjects, several primer pairs were designed on the longest contig obtained by NGS and its reverse complement sequence (Table 1). PCR was performed using a 25 µL reaction mix containing 5 µL of DNA, 1× PCR buffer, 2 mM MgCl_2_, 200 mM of dNTP, 0.8 mM of each primer, and 1.25 U of Taq polymerase (Invitrogen, Carlsbad, CA, USA). The thermal cycling conditions were 95 °C for 5 min; 45 cycles of 95 °C for 30 s, 58–60 °C for 30 s, and 72 °C for 30 s; and a final extension at 72 °C for 10 min. This PCR protocol was applied to brain (n = 3), skin (n = 1), and gill (n = 1) samples and pools of spleen, kidney, and heart tissue (n = 3) collected from diseased (n = 3) and aged-matched subjects (n = 2). In cases of positivity, PCR products of at least 2 diseased subjects and the aged-matched sturgeons were purified and subjected to sequencing. Sequences were obtained through the Bio-Fab Sequencing Service (Rome, Italy) and then compared with the sequence obtained by NGS.

## 3. Results

### 3.1. Pathological Findings

**Figure 2:**
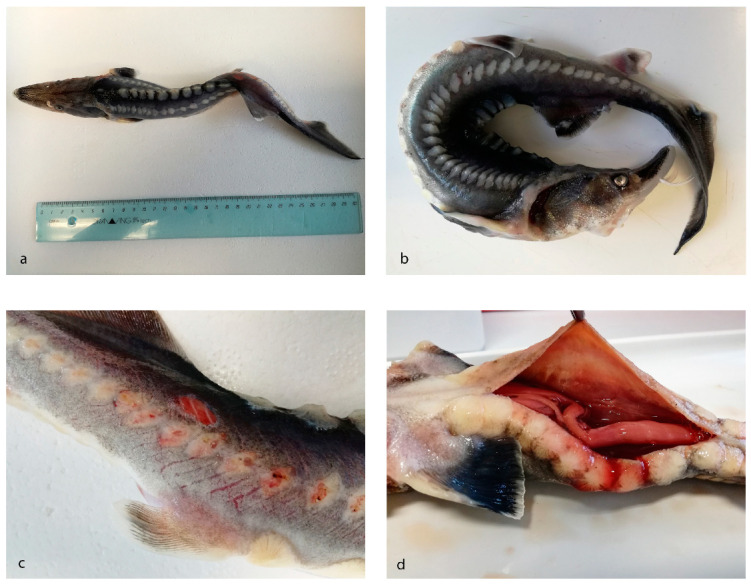
Gross examination *Huso huso*. (**a**,**b**) Two diseased animals showing body deformities and U-shaped body curvature. (**c**) Diseased animal showing ulcerative cutaneous erosions and reddening on the back. (**d**) Diseased animal showing a moderate amount of serohemorrhagic effusion in the coelomic cavity.

**Figure 3:**
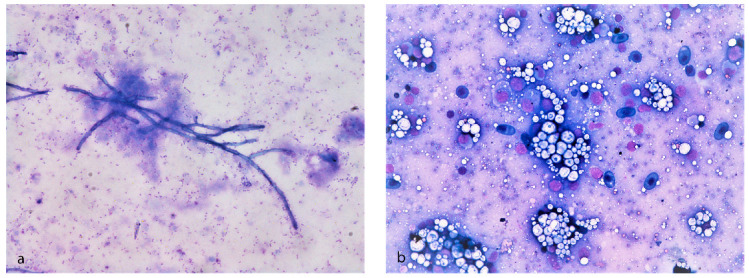
Cytological examination of *Huso huso* through May–Grünwald–Giemsa staining. (**a**) Cytologic smears from coelomic serohemorrhagic exudate showing several rod-shaped blue bacteria in the background and dark-blue branched septate hyphae. (**b**) Cytologic smears from kidney showing acellular, crystalline, material consistent with mineral deposit (nephrocalcinosis).

A total of 13 animals, 11 diseased and 2 aged-matched subjects, were examined. The length of the examined animals varied between 15 and 50 cm. Grossly, three subjects showed body deformities (Figure 2a,b), two of which also showed epaxial muscle softening. Three cases showed multifocal cutaneous reddening, erosions, and ulcerative dermatitis (Figure 2c). In two animals, a serohemorrhagic effusion in the coelomic cavity was present (1–1.5 cc) (Figure 2d). Smears from effusions contained a moderate amount of erythrocytes and cellular debris, admixed with several 1–2 µm rod-shaped blue bacteria. In one case, there were also numerous dark-blue-stained, septate hyphae 5–10 µm in transverse diameter with occasional branching (Figure 3a). PAS staining highlighted the fungal hyphae wall. In one animal, a chalky white, irregularly round and slightly elevated area a few millimeters in diameter within the renal parenchyma was sampled, and acellular, noncrystalline material consistent with a mineral deposit (nephrocalcinosis) was found (Figure 3b).

**Figure 4:**
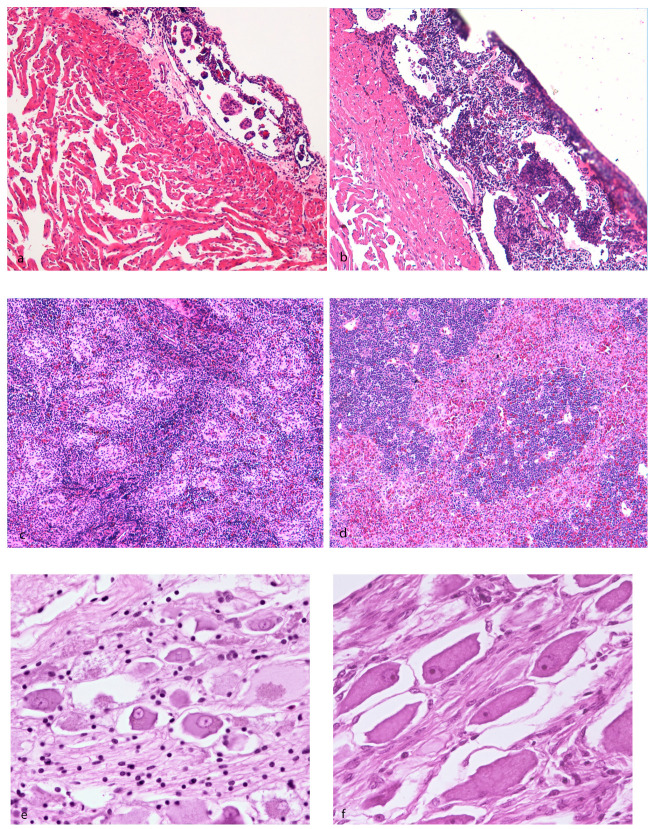

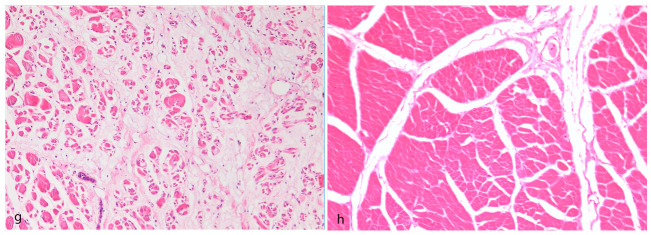
Hematoxylin–eosin staining of *Huso huso*. (**a**) Diseased animal showing the depletion of pericardial–epicardial lymphoid-like tissue compared to (**b**) aged-matched subject showing normal lymphoid tissue. (**c**) Diseased animal showing the depletion of lymphoid periarteriolar tissue and thickening of vessel wall (hyalinosis) compared to (**d**) aged-matched subject showing normal architecture. (**e**) Diseased animal showing the degeneration/atrophy of pyrenophores in the vicinity of epaxial muscles compared to (**f**) an aged-matched subject showing normal pyrenophores. (**g**) Diseased animal showing the degeneration and severe atrophy of skeletal myofibers and perimysial and endomysial edema compared to (**h**) an aged-matched subject showing normal skeletal muscle.

Histopathologic investigation revealed the depletion and rarefaction of hematopoietic tissue in seven cases, particularly the epicardial or renal or splenic tissue; in one case, depletion was simultaneously present in the three organs. Regarding the epicardium, a rather consistent finding among the evaluated subjects was the apparent prominence of the trabecular scaffold and a relative depletion of physiologic pericardial–epicardial lymphoid-like tissue (Figure 4a,b). Regarding the splenic parenchyma, arranged as mammalian red and white pulp [30], the red pulp appeared collapsed and the lymphoid periarteriolar sheaths contained moderately decreased numbers of lymphocytes; eosinophilic fibrillar material around the vessels and thickening of their walls was interpreted as a degeneration of the connective tissue (hyalinosis of the vessel wall) (Figure 4c,d). In the kidney (pronephros–mesonephros), discrete aggregates of hematopoietic tissue were interspersed among the excretory elements and islets constituting the normal interrenal tissues and glands. Within the lumina of the collecting tubules, discrete acellular basophilic mineralized foci were detected in two cases (nephrocalcinosis). In three cases, bacterial aggregates were detected within the vessels and brain. Viral inclusion bodies were not detected in the tissues examined.

In the investigated subjects, an inconstant decrease of interstitial lymphohematopoietic tissue was revealed; however, the actual amount was difficult to determine since, in sturgeon, there is a physiologic decrease of this component progressing through the caudal part of the organ. When available, the meningeal myeloid tissue, a unique hematopoietic site in sturgeon located over the medulla oblongata between the brain and the cartilaginous skull capsule [31], showed a mild reduction of erythroid, lymphoid, and granulocytic hematopoietic cells with a residual prominence of reticular stromal cells. In one case, transverse sections of intestine showed transmural colonization of the tissue, from the mucosal folds to the peritoneal serosa, by fungal hyphae 5–10 µm wide, septate, with irregular, dichotomous, and non-dichotomous acute-to-right-angle branching and with thin, pigmented, parallel walls (phaeohyphomycosis). In this case, a decrease in lymphoid cell aggregates and follicles, which are physiologically present in the connective tissue of the central typhlosole of the spiral valve [32], did not appear. In seven animals, the degeneration/atrophy of pyrenophores in the vicinity of the epaxial muscles was detected (Figure 4e,f). In the two animals displaying grossly muscular softening, multifocal vacuolar–hyaline degeneration of the myofibers ending in severe atrophy, with perimysial and endomysial edema, was revealed (Figure 4g,h); signs of regeneration and hypertrophy of the myofibers, such as rowing of nuclei and angular fibers, were not present.

### 3.2. Virus Investigation

Virological investigations produced negative results for all samples. Specifically, betanodaviruses, adenoviruses, and nucleocytoplasmic large DNA viruses were not detected using molecular methods (RT-PCR and PCR assays). With reference to *Herpesviruses*, their presence was assayed based on the detection of a generic PCR-targeting polymerase gene common to all large DNA viruses (including herpesviruses) and, notably, all samples tested negative [17]. No viral growth was observed on the WSSK cell line.

### 3.3. Bacteriological Examination

Bacteria were isolated from four out of six tested animals. Genetic characterization of the isolated bacteria showed the presence of *Aeromonas veronii*, *Shewanella putrefaciens, Chryseobacterium* sp., and *Citrobacter freundii*. In particular, *A. veronii* and *S. putrefaciens* were isolated from brain, kidney, spleen, and skin lesions, *Citrobacter freundii* was isolated from the kidney and brain, and *Chryseobacterium* sp. was isolated from brain and skin lesions, confirming bacterial septicemia.

*A. veronii* colonies appeared white, convex, and circular on the TSA medium and were composed of Gram-negative bacteria. Sequencing of the gyrase subunit beta (*gyr*B) and 16S rDNA gene fragment confirmed the affiliation of putative *A. veronii* isolates with *A. veronii* reference strain ATCC 35624 with a similarity of 98.1–98.5% and 99.6–100% with GenBank accession numbers AF417626 and NR118947, respectively. *S. putrefaciens* colonies appeared pigmented, convex, and circular on the TSA medium and were composed of Gram-negative bacteria. Sequencing of the 16S rDNA gene fragment confirmed the affiliation of these isolates with *S. putrefaciens* reference strain ATCC 8071 (GenBank accession number X82133) with a similarity of 98.1%. *C. freundii* colonies appeared mildly pigmented, convex, and circular on the TSA medium and were composed of Gram-negative bacteria. Sequencing of the 16S rDNA and *gyr*B gene fragments confirmed the affiliation of this isolate with *C. freundii* reference strain ATCC 8090 (GenBank accession number AJ300528) with a similarity of 99.8% and 97.5% with GenBank accession number NR_028894 and AJ300528, respectively. *Chryseobacterium* sp. colonies appeared as small yellow colonies on the TSA medium and were composed of Gram-negative bacteria. Sequencing of the 16S rDNA gene fragment confirmed the affiliation of putative *Chryseobacterium* sp. isolates with a *Chryseobacterium* sp. strain previously isolated from white sturgeon in the United States (GenBank accession number MK614007) with a similarity of 99.2%.

The bacterial sequences obtained in this study were deposited into the GenBank database and are available under the following accession numbers MW080649-MW080655, MW086625- MW086628.

### 3.4. Chemical Investigations

Selenium concentrations in the hearts of diseased animals were significantly lower than those observed in age-matched animals (0.747 and 0.995 vs. 1.759 and 2.033 mg/kg) (*t* test; *p* = 0.031).

### 3.5. NGS and Scaffold Analysis

Illumina sequencing produced a total of 92,883,444 paired-end reads that were 151 bp long. After quality filtering, we retained 84,828,159 paired-end reads (91.3%) with an average length of 139 bp.

Metagenomic analysis of high-quality data showed that the vast majority of reads (>99.9%) belonged to the host *Huso huso* or were impossible to taxonomically classify, since the complete genome sequence information for *Huso huso* is still lacking. Only a small fraction of reads (0.05%) was found to belong to bacteria (mainly *Aeromonas veronii* and *Shewanella putrefaciens)*. Viral reads comprised an even smaller fraction, with only 1485 high-quality reads classified as belonging to the *Herpesvirales* order.

De novo assembly of these *Herpesvirales* reads produced 13 contigs between 352 and 1167 bp long (average 582 bp). Most of them did not show any similarity with known herpesvirus sequences, with a couple of exceptions as represented by the third-longest contig (740 bp, 68% similarity with herpesvirus terminase) and the longest contig. The latter, despite having poor query cover (3%) but high similarity (89%) with herpesvirus DNA-dependent DNA polymerase at the nucleotide level, showed high query coverage at the amino acid level (87%) and enough similarity (46%) with DNA polymerase to be considered as a putative herpesvirus DNA polymerase (Table S1).

The MiSeq raw data were submitted to the NCBI Sequence Read Archive (SRA; www.ncbi.nlm.nih.gov/Traces/sra/) under accession number SRR12532268.

PCR assays conducted with primers designed against putative herpesvirus DNA polymerase and its reverse complement sequence permitted us to obtain specific PCR products from all diseased and age-matched sturgeons (data not shown). Sequence analysis showed 100% nucleotide identity between sequences obtained from diseased subjects and age-matched sturgeons, as well as with the sequence of the longest contig obtained by NGS (data not shown).

## 4. Discussion and Conclusions

In examining the diseased sturgeons, the clinical signs that most caught our attention were the behavioral alterations, particularly whirling and uncoordinated swimming movements. This finding is reminiscent of what was reported in *A. baerii* juveniles, which similarly displayed neurological signs such as lethargy, inability to maintain an upright position, and erratic swimming [6]. In that case, the authors found the presence of Acipenser iridovirus European (AcIV-E), a virus included among sturgeon NCLDVs, and concluded that stressful transport conditions probably played an important role in exacerbating the mortality [6]. In our case, chronic dripping mortality lasting several months was registered, but it was not associated with movement/handling. Moreover, virological investigation excluded the presence of any of the known sturgeon NCLDVs. Furthermore, the sturgeons’ neurological signs were associated with body deformity represented by arching of the back and vertebral silhouette deformation. Since they were housed in tanks employed for other aquatic species and considering their high susceptibility to even minimal environmental changes, several factors, including acoustic phenomena due to the hydraulic and housing systems, could have contributed to disrupting axial architecture homeostasis.

The differences in the vertebral structure between fish with an unconstricted notochord, such as Acipenseriformes, and fish with vertebral centra, such as teleosts, are correlated with different swimming types and, thus, different mechanical constraints on the vertebral column and the notochord could have had an effect on the emergence of axial anomalies. Nonetheless, the causes of these pathologies are poorly documented [33]. Athanassoupoulou et al. [34] reported that juvenile sturgeons reared in open-flow systems were susceptible to skeletal abnormalities of the spine such as scoliosis and lordosis. Histopathological studies were inconclusive concerning etiology, but anomalies seemed to be related to the horizontal transmission of betanodavirus by infected sea bass. Clinical signs of benodavirus infection are neurologic [35,36] and consist of abnormal, corkscrew swimming [37], and erratic swimming in descending circles, which can cause curvature of the dorsal spine [38]. In the cases described here, infection due to *Betanodavirus* was initially highly suspected due to the clinical neurological presentation, but microscopic and molecular investigations excluded this possibility.

Another severe gross change was represented by epaxial muscle softening, which histologically matched with multifocal degeneration progressing into myofiber atrophy and interstitial edema. The damage to skeletal muscles was partly explained by the sensory deficit (degeneration and atrophy of gangliar neurons close to the skeletal myofibers) as the basis of motor deficit. Knowledge about skeletal muscle histology and its pathological counterpart in sturgeon is limited: studies are focused on myogenesis during embryonic and larval development growth and correlation between growth rate and muscle development in fast- and slow-growing fingerlings [39]. On the basis of the bacteriological results, the putative role of *Shewanella* spp. as a pathogen with a primary role in developing muscular changes was taken into consideration, as also suggested in koi carp showing generalized edema, among other clinical signs [40]. In this case, the authors supposed that the habitat of these bacteria is probably expanding to different freshwater ecosystems, including ornamental species.

Although aquaculture for sturgeon production has increased worldwide, infectious diseases are one of the major factors still limiting its expansion [41]. Literature on bacterial pathogens in sturgeons is scarce, probably because these animals seem quite resistant to diseases after they reach a certain size [42]. Regarding *Huso huso* and its hybrids, outbreaks reported in relation to bacteria have referred to *Vibrio vulnificus*, *Aeromonas hydrophila* and *A. veronii*, *Lactococcus lactis*, and *Yersinia ruckeri* [10,11,43,44]. Kayiş et al. [42], during a three year-period of observation, isolated several bacteria, helping to create a profile of those to which the reared sturgeons are susceptible. However, most of the bacteria isolated from sturgeons are opportunistic, and the bacteria are often reported as secondary infections or as a consequence of severe stress and high stock density [2,11]. Recently, a report of *Staphylococcus iniae* in *A. transmontanus* was published. Bacterial cocci were found scattered throughout muscle lesions, highlighting the emergence of necrotizing and heterophilic myositis in cultured sturgeon. However, other factors such as fish movement, increased stocking density, and increased water temperature suggested important epidemiologic factors [45].

The presence of ulcerative dermatitis and serohemorrhagic coelomic effusions in the studied cases easily correlate with the bacteriological findings, showing bacterial septicemia due to several species such as *A. veronii, S. putrefaciens*, *Citrobacter freundii*, and *Chryseobacterium* sp. Variable pathogenicity against sturgeons was reported for the detected bacteria. Motile *Aeromonas* infection is one of the most common infections in sturgeons [11]; however, only a few cases have actually documented the isolation of *A. veronii* from diseased sturgeons [10,46]. Particularly, in stellate sturgeon (*Acipenser stellatus*), co-infection due to *A. veronii* and *Chryseobacterium joostei* has been reported [10]. *Chryseobacterium* sp. has been isolated from diseased sturgeons, including the species *Huso huso*, in combination with ulcerative skin lesions, ascites, and increased mortality [10,47]. However, experimental infections with *Chryseobacterium* sp. isolated from fish were not able to induce any clinical signs or gross pathological changes in white sturgeon [47], diverging from what was previously observed in other fish species with other *Chryseobacterium* sp. strains [48]. In addition, *Citrobacter freundii* has been previously isolated from the internal organs of diseased sturgeons, but no detailed description has been reported regarding these infections [42]. On the other hand, to the best of our knowledge, *S. putrefaciens* has not yet been associated with diseased sturgeons; however, this bacterium has been frequently isolated from diseased fish during mortality outbreaks and is associated with septicemia [49,50]. The presence of co-infections found in our study and the general body condition outlined by the pathological findings argue for a secondary role of these bacterial infections, leading us to conduct further investigations to elucidate the primary cause.

In this study, the combination of the young age of the sturgeons and the muscular findings is reminiscent of white muscle disease, a degenerative myopathy of mammals and poultry in which the skeletal muscle, heart, and gizzard, in the case of birds, are the predilection sites. Striated muscles and heart undergo waxy degeneration, which disrupt the architectural features and provide a whitish color. In young animals, white muscle disease is the most prevalent disorder resulting from selenium deficiency; the first organs affected are the heart, skeletal muscle, and liver [51].

Considering these literature findings in other species and the evidence for epaxial muscle softening in some of the studied sturgeons, frozen heart tissue from a selection of diseased animals and age-matched juveniles was analyzed using ICP–OES to determine the selenium concentration. Despite the low number of tested samples and the absence of reference values from the literature, the significantly lower concentration of selenium found in diseased animals reinforces the nutritional pathogenesis of this muscle change as observed in the present study. Among the clinicopathological presentations of selenium deficiency, exudative diathesis, represented by increased capillary permeability, edema, swelling, and bruising, is reported in poultry and pigs [51], similar to the examined sturgeons. Studies on nutrient requirements and utilization, conducted under laboratory conditions, provided evidence that the dietary selenium requirement for juvenile beluga sturgeon is between 11.56 and 20.26 mg Se/kg diet when Se supplementation is in the form of L-selenomethionine. Such nutrient requirements and utilization, and even data on general nutrition, are needed for success in rearing these species [52]. Furthermore, selenium can be found in large amounts in the spleen, liver, and lymph nodes, where it contributes to stimulating antibody formation and T helper cell activity along with that of cytotoxic T and NK cells, phagocytic cell migration, and phagocytosis. Thus, the sampling of sturgeon heart also included lymphoid-like cells, since they possess unique lymphomyeloid structures situated in the pericardium. Based on the results showing reduced concentrations of selenium, it is hypothesized that in the diseased sturgeons, immune functions could possibly have been depressed as a response to selenium decrease. Indeed, the most significant histopathologic findings regarding the lymphohematopoietic tissues (spleen and pericardial lymphoid tissue, in particular) were the depletion and rarefaction of cells. The sturgeon’s splenic parenchyma normally consists of white pulp arranged in follicle-like periarterial cells and red pulp; ellipsoidal arterioles are surrounded by red pulp [30,53]. In some observed cases, the red pulp was instead devoid of cells, the white pulp was depleted, and the follicle-like arrangement was lost. The cranial kidney, composed exclusively of hematopoietic tissue and islets of interrenal tissue [52], also showed a reduction in hematopoietic cells. The meningeal myeloid tissue (also called hemopoietic focus), a bone marrow-like structure typical of sturgeon [30], was not routinely sampled in our study, but, where present, it also appeared depleted. The reduction in renal hematopoietic tissue is generally difficult to assess in sturgeons due to its physiologic reduction when proceeding caudally [54]; therefore, the standardized sampling of different areas of the kidney should be planned for the proper quantification of hematopoietic tissue.

All of these findings lead to the supposition of chronic wasting disease, which has been reported in the literature in sturgeon associated with adenovirus infection [55]. Sturgeon wasting disease is a chronic disease of cultured white sturgeon that was first described in 1985 [56]. Fry showed lethargy, anorexia, emaciation, and severe mortality. The gross pathology consisted of pale liver and no food in the intestinal tract, and adenovirus infection was confirmed by electron microscopy [55]. Sturgeon adenovirus was later isolated and genetically characterized, and a molecular method to detect it has been developed [15,55]. The molecular method used by these authors to detect sturgeon adenovirus was applied in our study [15], in addition to a consensus nested-PCR method designed for investigating a broad range of adenoviruses that is useful for obtaining templates for the initial sequencing of novel adenoviruses [16], and both methods confirmed the absence of this etiological agent.

Among fish viral pathogens, herpesviruses are also known to cause the destruction of hematopoietic tissues [57]. To investigate the presence of herpesvirus in the affected fish, a broadly applicable method to characterize large DNA viruses, including herpesvirus, was applied [17] but no herpesvirus-specific products were obtained with this method. On the other hand, NGS allowed the acquisition of a sequence consistent with the herpesvirus DNA polymerase gene. However, further investigation of this fragment showed its ubiquitous presence in analyzed sturgeons, including both diseased and aged-matched subjects. Even though further study is needed to fully understand the nature and role of this sequence, from our investigation, it seems it was not correlated with the mortality outbreak herein described.

Pathological investigation also showed splenic vessel hyalinosis and nephrocalcinosis, which were interpreted as a tissue response to suboptimal environmental factors. In fact, studies on the morphofunctional state of mesonephros in various sturgeons and bony fishes species taken from natural waters recorded different changes in structural–functional elements of nephrons, such as hemorrhages in the intertubular tissue of the kidney, hyalinosis of splenic vessel walls, swollen epithelium, and inflammation, which were interpreted as consequences of environmental conditions [58].

The systemic phaeohyphomycosis that was cytologically and histologically diagnosed in one sturgeon could reinforce the hypothesis of suboptimal environmental conditions and stressors. Among pigmented mycoses, *Veronaea botryosa* causes systemic infections in reared sturgeons, but little is known regarding the environmental conditions that lead to this infection. It was demonstrated that sturgeon maintained at 18 °C developed systemic phaeohyphomycosis and had significantly greater mortality than controls and fish maintained at 13 °C [9].

In conclusion, this multidisciplinary investigation points out several pathological findings associated with the described chronic mortality outbreak. In the absence of a primary yet undetected biological cause, it is supposed that environmental stressors, including nutritional imbalances, may have led to immune system impairment, facilitating the entry of opportunistic bacteria and mycotic hyphae. Septicemia, responsible for cutaneous changes and serohemorrhagic coelomic effusion, grossly prevailed. As a whole, the histopathological findings are consistent with immune system impairment, and the degenerative changes reflect a negative response to a suboptimal environmental state, so the musculoskeletal changes may be considered to be the effect of circulatory and nutritional imbalances.

However, the absence of solid knowledge and available data regarding the pathology, nutrition, physiology, and microbiology of these challenging animals undermines the full understanding of all observations. In this respect, this study provides novel data regarding the pathology, chemistry, and molecular biology of one of the most important sturgeon species, *Huso huso*. Overall, these findings provide scientific foundation for the interpretation of future case studies and toward overcoming the challenges inherent in the farming of this and other related species.

## Figures and Tables

**Table 1:** Primers used to investigate putative herpesvirus DNA polymerase sequence obtained through next-generation sequencing (NGS).

Primers	Sequence	Scaffold Orientation	Amplicon (bp)
P1_for	agcctggaacggtatctg	3′→ 5′	273
P1_rev	tacggtgctgtcggt		
p1tq_for	ctttccgcaagcgcagatac	5′→ 3′	331
p1tq_rev	ttcgtcggcggatacgttag		
p1cr_for	ttcgtcggcggatacgttag	3′→ 5′	331
p1cr_rev	cgctgccatcgatttgtgag		
P3tq_for	taacgtatccgccgacgaag	5′→ 3′	325
P3tq_rev	acctccctgtaccgctactt		
P3cr_for	acctccctgtaccgctactt	3′→ 5′	325
P3cr_rev	taacgtatccgccgacgaag		

## Supplementary Materials

The following are available online at https://www.mdpi.com/2076-2615/10/10/1866/s1, Table S1, NGS BLAST analysis results. Video S1, neurologic signs observed in diseased sturgeons.
