# Supplementary material for: Multifactorial Causes of Chronic Mortality in Juvenile Sturgeon (Huso huso)
Source: Animals (Basel). 2020 Oct 13;10(10):1866. doi: 10.3390/ani10101866 (PMC7602020; doi:10.3390/ani10101866)
Supplement: Supplementary file 1 [file animals-10-01866-s001.zip › Table S1-Supplementary_file - final.docx]

| **Contig** | **Length (bp)** | **BlastN best hit result** | | | | **BlastX best hit result** | | | |
| --- | --- | --- | --- | --- | --- | --- | --- | --- | --- |
|  |  | **Acc. Num.** | **E-value** | **Query cover** | **Similarity** | **Acc. Num.** | **E-value** | **Query cover** | **Similarity** |
| scaffold_0 | 1167 | KM357278.1 | 6e-04 | 3% | 88.89% | RXM96545.1 | 0.0 | 99% | 99.23% |
| scaffold_1 | 884 | XM_030554500.1 | 0.25 | 3% | 100% | RXM96537.1 | 4e-177 | 97% | 98.8% |
| scaffold_2 | 740 | EF535575.1 | 8e-13 | 33% | 67.74% | RXM96558.1 | 9e-51 | 44% | 97.27% |
| scaffold_3 | 647 | LR633957.1 | 0.62 | 4% | 96.55% | YP_009362373.1 | 2e-64 | 96% | 47.42% |
| scaffold_4 | 620 | CP050504.1 | 0.6 | 5% | 93.94% | RXM91269.1 | 1e-20 | 22% | 97.87% |
| scaffold_5 | 549 | CP000830.1 | 0.52 | 8% | 84.09% | YP_656588.1 | 1e-17 | 66% | 38.52% |
| scaffold_6 | 472 | CP051010.1 | 0.13 | 5% | 100% | YP_003358243.1 | 1e-11 | 73% | 39.66% |
| scaffold_7 | 469 | CP025228.1 | 0.44 | 6% | 93.55% | NP_041135.1 | 1e-22 | 82% | 38.24% |
| scaffold_8 | 454 | DQ665652.1 | 0.43 | 9% | 84.09% | RXM96561.1 | 4e-108 | 99% | 100% |
| scaffold_9 | 446 | MH048901.1 | 2e-05 | 26% | 72.88% | RXM96544.1 | 5e-94 | 99% | 99.32% |
| scaffold_10 | 413 | LN829119.1 | 1.3 | 6% | 100% | RXM96545.1 | 3e-77 | 99% | 98.54% |
| scaffold_11 | 357 | LR132053.1 | 4.0 | 25% | 82.61% | - | - | - | - |
| scaffold_12 | 352 | LR738420.1 | 0.32 | 9% | 91.67% | RXM96556.1 | 1e-60 | 86% | 99.02% |
